# Eye-Tracking Analysis of Visual Attention and Preference in Human-Created, AI-Generated, and AI-Refined Human Advertisements

**DOI:** 10.3390/s26206411

**Published:** 2026-10-10

**Authors:** André Gonçalves, Margarida Afonso, Pedro Martins, Ana Teixeira

**Affiliations:** 1 Instituto Superior de Engenharia do Porto (ISEP), Rua Dr. António Bernardino de Almeida 431, 4249-015 Porto, Portugal; 1230794@isep.ipp.pt; 2 Research Group on Intelligent Engineering and Computing for Advanced Innovation and Development (GECAD), Instituto Superior de Engenharia do Porto (ISEP), Rua Dr. António Bernardino de Almeida 431, 4249-015 Porto, Portugal; afo@isep.ipp.pt; 3 Coimbra Business School (ISCAC), Polytechnic University of Coimbra, Rua da Misericórdia, Lagar dos Cortiços, S. Martinho do Bispo, 3045-093 Coimbra, Portugal; pmmartins@iscac.pt; 4 Center for Research and Innovation in Education (InED), School of Education, Polytechnic of Porto, Rua Dr. Roberto Frias 602, 4200-465 Porto, Portugal

**Keywords:** eye tracking, generative artificial intelligence, visual attention, advertising, AI-refined advertising, visual perception, consumer choice

## Abstract

Generative artificial intelligence (GenAI) is increasingly used in advertising, yet its effects on visual attention and preference remain insufficiently characterized. This study compared human-created (H), AI-generated (AI), and AI-refined human (HAI) advertisements, the latter obtained by submitting human-created advertisements to a generative model with a standardized refinement prompt, using eye tracking and a forced-choice task. Thirty-nine young adults (18–26 years) with high generative-AI experience completed 858 trials involving 22 advertisement pairs while gaze was recorded at 60 Hz. Fixation duration, fixation count, time to first fixation, saccadic magnitude, and pupil diameter were compared using repeated-measures analyses of participant-level means with Holm correction, and choices were analyzed using binomial models with participant-clustered standard errors. AI-generated advertisements were selected more frequently than human-created advertisements (58.97% of trials; odds ratio = 1.44, p=0.0015), whereas HAI advertisements did not differ from human-created advertisements in choice frequency (49.88%; p=0.978). Relative to human-created advertisements, both AI and HAI stimuli were associated with higher fixation counts and smaller saccadic magnitudes, with these effects surviving Holm correction. Time to first fixation was statistically equivalent across conditions within the smallest effect detectable by the design, pupil diameter did not differ, and differences in fixation duration did not survive correction. The higher fixation counts reflected a larger share of fixation time on AI and HAI advertisements rather than a higher fixation rate. Although the gaze pattern was similar for AI and HAI stimuli while only AI stimuli were preferred, the difference between the two choice effects was not statistically reliable. The observed gaze differences cannot be uniquely attributed to creative origin because AI and HAI stimuli also exhibited higher edge density than human-created stimuli. These findings support the use of eye tracking as a sensor-based method for characterizing visual responses to AI-assisted advertising while highlighting the need for tighter stimulus control and more diverse samples in future studies.

## 1. Introduction

The rapid advancement of generative artificial intelligence (AI) has fundamentally reshaped the landscape of digital content creation, particularly within marketing and advertising ecosystems. Contemporary generative models—such as diffusion-based image generators and large-scale multimodal systems—enable the automated production of highly realistic and visually compelling advertisements at unprecedented scale. This technological shift introduces both opportunities and challenges, particularly concerning how human users perceive, process, and respond to AI-generated and co-created visual stimuli.

Understanding visual attention is central to evaluating the effectiveness of advertising content. Eye-tracking technology provides an objective and fine-grained method for capturing attentional processes by measuring gaze behavior through metrics such as fixation duration, fixation count, saccadic transitions, pupillary response, and blink dynamics [1,2,3]. Eye-tracking provides objective measures of visual behavior, including fixation duration, fixation count, time to first fixation, saccadic movements, blink behavior, and pupil responses. These measures can provide indirect evidence about visual exploration, attentional allocation, and processing demands, although their interpretation depends strongly on task context and stimulus properties. In recent years, eye-tracking has become a key methodological tool in neuromarketing, human–computer interaction, and cognitive science, enabling the quantification of user engagement beyond self-reported measures [4,5].

Concurrently, emerging research has begun to investigate how AI-generated content affects human perception and cognition. Recent studies indicate that users exhibit distinct gaze patterns when exposed to synthetic versus human-created visual stimuli, often characterized by increased scrutiny, altered fixation distributions, and variations in trust-related processing [6,7,8]. For instance, experimental evidence suggests that AI-generated images may trigger longer or more dispersed visual exploration due to uncertainty regarding authenticity, while human-created content tends to facilitate more efficient visual processing and stronger perceptual coherence. Furthermore, the integration of physiological sensing and eye-tracking has revealed that AI-generated media can modulate cognitive load and affective responses, particularly in contexts where realism and credibility are critical.

An adjacent literature establishes that evaluations of AI-authored creative work depend strongly on whether the audience is told the origin of the work. Disclosure of machine authorship has been shown to reduce appreciation of visual art and to depress evaluations of algorithmic output more generally [9,10,11,12,13]. This boundary condition is directly relevant to the present design, in which stimulus origin was never disclosed to participants, and it is returned to in Section 5.

Despite these advances, a significant research gap persists. Existing studies predominantly focus on detection accuracy, authenticity judgment, or general perception of AI-generated content, rather than systematically examining how visual attention mechanisms differ in applied contexts such as advertising. Moreover, the relationship between gaze behavior and choice behavior in AI-mediated environments remains insufficiently explored. In particular, there is a lack of controlled experimental studies that directly compare AI-generated, human-created, and AI-refined advertisements using objective behavioral metrics and statistically robust methodologies. Although previous studies have examined the perception and evaluation of AI-generated visual content, comparatively less attention has been given to controlled comparisons of human-created, fully AI-generated, and AI-refined human advertising stimuli using eye-tracking measures while participants make preference decisions. In particular, it remains unclear whether differences in gaze behavior associated with AI-assisted visual production are accompanied by corresponding differences in behavioral preference. To address this gap, the present study investigates how visual attention differs between AI-generated, human-created, and AI-refined human (HAI) advertisements and how these differences relate to user preference. Four hypotheses were evaluated:

**H1.**  *Relative to the human-created stimulus set, the AI-generated and AI-refined stimulus sets are associated with a higher fixation count.*

**H2.**  *Relative to the human-created stimulus set, the AI-generated and AI-refined stimulus sets are associated with smaller saccadic magnitudes.*

**H3.**  *Time to first fixation is equivalent, within the smallest effect detectable by the design, for AI-generated, AI-refined, and human-created advertisements.*

**H4.**  *Differences in eye-tracking measures do not consistently correspond to differences in forced-choice preference across the AI-versus-human and AI-refined-versus-human contrasts.*

H1 and H2 are formulated as associations at the level of the stimulus sets rather than as effects of creative origin, because origin was not experimentally separated from low-level structural properties of the images (Section 3.1). H3 is formulated as an equivalence hypothesis and is evaluated with equivalence tests and Bayes factors rather than with a non-significant *p*-value. The hypotheses were not preregistered, and their present wording was established after the data had been collected and analyzed. In particular, the formulation of H1 and H2 as associations with the stimulus sets and of H3 as an equivalence hypothesis was adopted after the analysis of the data. The corresponding tests therefore evaluate the hypotheses as stated here but should not be read as tests of predictions fixed before data collection.

The main contributions of this paper are as follows:

- A controlled experimental comparison of AI-generated vs. human-created advertisements and AI-refined vs. human-created advertisements using eye-tracking metrics;
- A statistical analysis linking gaze behavior to choice behavior, with correction for multiple comparisons and reported effect sizes;
- An empirical characterization of the attentional differences associated with synthetic, AI-refined, and human-authored visual content under non-disclosure of stimulus origin;
- Evidence informing the interpretation of eye-tracking responses to AI-assisted advertising stimuli and their relationship with forced-choice preference.

The remainder of this paper is organized as follows. Section 2 reviews the relevant literature on eye tracking, visual attention, and AI-generated content. Section 3 describes the experimental methodology, including participants, apparatus, and metrics. Section 4 presents the results of the statistical analysis. Section 5 discusses the findings and their implications for theory and practice. Section 6 states the limitations of the study. Finally, Section 7 concludes the paper and outlines directions for future research.

## 2. Related Work

Understanding how humans visually process advertising stimuli requires a multidisciplinary perspective that integrates cognitive science, visual perception, and sensing technologies. In this context, eye-tracking has emerged as a fundamental methodological tool, enabling the objective quantification of attention allocation through high-resolution gaze data. Simultaneously, the proliferation of generative artificial intelligence (AI) has introduced a new class of visual stimuli whose perceptual and cognitive processing mechanisms remain only partially understood.

Recent literature has increasingly focused on the intersection between AI-generated content and human perception, highlighting differences in attention patterns, trust formation, and cognitive load. However, these research streams have largely evolved in parallel. On the one hand, eye-tracking studies have extensively characterized visual attention in marketing and interface design; on the other hand, AI-focused research has primarily examined issues of authenticity, realism, and detectability. The integration of these domains—particularly in applied contexts such as advertising—remains underdeveloped.

This section reviews the state of the art across three key dimensions: (i) the role of eye tracking in analyzing visual attention within marketing contexts, (ii) the perceptual and cognitive implications of AI-generated visual content, and (iii) emerging efforts to integrate AI methodologies with eye-tracking data for enhanced behavioral analysis.

### 2.1. Eye Tracking in Marketing and Visual Attention

Eye-tracking has become a central tool in the quantitative analysis of visual attention in marketing and advertising research. Metrics such as fixation duration and fixation count are strongly associated with cognitive processing, attentional engagement, and information encoding [3,4]. Empirical studies consistently demonstrate that visually complex and dynamic stimuli elicit increased fixation density and prolonged gaze durations, reflecting higher levels of cognitive involvement and attentional capture [14].

Beyond descriptive analysis, eye-tracking has been widely applied to evaluate advertising effectiveness, optimize user interfaces, and assess visual hierarchy [5,15,16,17]. These studies reveal that visual saliency, spatial layout, and design structure play a critical role in guiding attention distribution and shaping user engagement [18]. Importantly, gaze-based metrics have been shown to correlate with higher-level behavioral outcomes, including recall, preference, and decision-making, reinforcing their value as objective indicators of consumer response [19,20].

Two findings from this literature bear directly on the present design. First, in binary preference tasks, gaze progressively shifts toward the eventually chosen alternative in the seconds preceding the decision—the gaze cascade effect [19]—so that dwell-based measures and choice are mechanistically coupled rather than independent [20]. Any comparison of fixation measures between two simultaneously presented alternatives must therefore be interpreted in light of this coupling. Second, low-level image statistics alone predict a substantial share of early fixation placement [18], which makes explicit stimulus matching a precondition for attributing gaze differences to creative origin.

### 2.2. AI-Generated Content and Human Perception

The rapid adoption of generative AI systems has introduced new challenges in visual perception, particularly regarding authenticity, trust, and cognitive processing. Recent evidence suggests that users often struggle to reliably distinguish AI-generated content from human-created stimuli, with classification accuracy frequently falling below 80% in controlled experimental settings [21]. This perceptual ambiguity has important implications for attention allocation and cognitive load.

Eye-tracking studies indicate that perceived authenticity modulates gaze behavior. Specifically, when users suspect that content may be AI-generated, they tend to exhibit increased visual scrutiny, characterized by longer fixation durations and more dispersed scanpaths [6,7]. Such patterns suggest heightened cognitive effort associated with verification and uncertainty resolution.

Two recent studies are especially close antecedents of the present work. de Winter et al. [6] recorded gaze while participants judged whether images were Midjourney-generated, and reported that discrimination performance and gaze allocation depend on the presence of localized generation artefacts rather than on global image properties. Kiper and Liang [22] compared AI-generated and real fashion advertisements with eye-tracking and found broadly comparable attentional outcomes across origins. The present study differs from both in that origin was never disclosed or queried, and in that a hybrid human–AI condition is included alongside the fully synthetic one.

Moreover, recent multimodal studies combining eye tracking with physiological sensing have demonstrated that AI-generated content can influence trust formation in complex ways, depending on contextual cues such as labeling and disclosure [23,24]. These findings underscore the importance of transparency and design strategies in mitigating potential negative effects associated with synthetic media [9,11].

### 2.3. Integration of AI and Eye Tracking

The integration of AI techniques with eye-tracking methodologies represents a promising direction for advancing behavioral analysis and adaptive system design. Eye-tracking provides fine-grained, real-time measurements of user attention, while AI enables scalable data processing, pattern recognition, and predictive modeling [25,26]. Together, these technologies offer the potential to develop intelligent systems capable of dynamically adapting content based on user gaze behavior [27].

However, despite this potential, comprehensive studies that systematically combine AI-generated stimuli with eye-tracking analysis in applied domains such as marketing remain limited [28]. Existing work has primarily focused on either optimizing gaze prediction models or analyzing perception of AI-generated media in isolation, rather than integrating both perspectives within a unified experimental framework [29].

Additionally, the convergence of AI and eye tracking raises important ethical considerations, including risks related to behavioral manipulation, privacy, and data governance. The use of gaze data as an implicit behavioral signal introduces concerns regarding user autonomy and informed consent, particularly in commercial applications [27,30]. Addressing these challenges is essential for the responsible deployment of AI-driven attention-aware systems.

### 2.4. Research Gap and Study Rationale

Taken together, the literature suggests that three processes may be relevant to the evaluation of AI-assisted advertising: the visual properties of the stimulus, the allocation of visual attention, and the subsequent preference decision. These processes should not be assumed to be equivalent. Low-level image properties can influence early gaze allocation, while gaze can also become coupled to choice during evidence accumulation. At the same time, knowledge about AI authorship may influence evaluative judgments independently of visual processing. The present study therefore focuses on the empirical relationship between these components under a controlled non-disclosure condition. By comparing human-created, fully AI-generated, and AI-refined human advertisements, the study asks whether differences in gaze behavior are consistently accompanied by differences in forced-choice preference.

## 3. Methodology

The methodology is organized into six main sections: an overview, the experimental design, participant characteristics, data acquisition, the experimental procedure, and data processing and statistical analysis.

### 3.1. Overview

This study adopts a within-subject experimental design to examine differences in visual attention across advertising stimuli with distinct creative origins. Specifically, the analysis compares human-created, AI-generated, and AI-refined human advertisements. By exposing each participant to all stimulus conditions, the design enables direct comparison while controlling for inter-individual variability.

**Figure 1:**
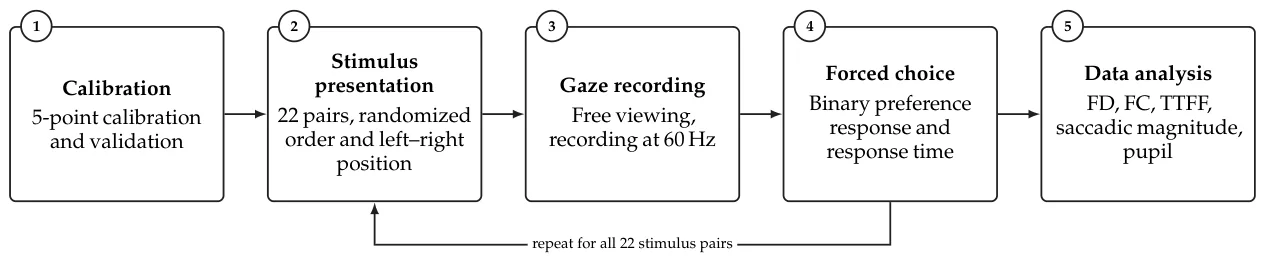
Experimental workflow. Each session comprised a 5-point calibration, followed by 22 advertisement pairs presented in randomized order with randomized left–right placement. Gaze was recorded at 60 Hz during free viewing of each pair, after which participants made a binary forced choice. Steps 2–4 were repeated for all 22 pairs; the eye-tracking metrics defined in Section 3.6 were computed offline. No fixation cross or drift check was presented between pairs.

To empirically evaluate stimulus comparability, low-level image statistics were extracted across conditions. All statistics were computed in Python with OpenCV on the grayscale version of each advertisement: mean luminance as the mean pixel intensity (0–255), RMS contrast as the standard deviation of pixel intensities, edge density as the mean value of the binary Canny edge map (0–255 scale), and saliency dispersion as the spatial standard deviation of the spectral-residual saliency map. Synthetic and human versions were compared with paired *t*-tests across the 11 brands (df=10). Paired comparisons showed no significant differences in mean luminance (t(10)=−0.18, p=0.861), RMS contrast (t(10)=−0.65, p=0.531), and computational saliency dispersion (t(10)=1.72, p=0.115). However, quantitative differences emerged in structural complexity: edge density was higher for AI-generated than for human-created stimuli ($M_{\mathrm{AI}}=59.09$ vs. $M_{H}=40.73$; t(10)=3.58, p=0.005) and higher for AI-refined than for human-created stimuli ($M_{\mathrm{HAI}}=61.56$ vs. $M_{H}=40.73$; t(10)=8.26, p<0.001), each tested with a paired *t*-test across the 11 brands. Because only 11 brand pairs were compared, the non-significant tests indicate the absence of large imbalances rather than demonstrated equivalence. The complete experimental workflow is illustrated in Figure 1.

### 3.2. Experimental Design

Participants were exposed to a total of 22 pairs of advertisements, distributed across three stimulus categories:

- **Human-created (H):** Advertisements fully designed by human creators;
- **AI-generated (AI):** Advertisements generated autonomously using generative AI systems, from detailed brand-specific text prompts that described the content of the corresponding campaign;
- **AI-refined human (HAI):** Human-created advertisements that were uploaded to a generative model together with a single standardized instruction to make the image more appealing (Table S1). The human contribution in this condition is the source design; the modification itself was performed by the model without further human guidance.

All AI-generated and AI-refined images were produced with the native image-generation capability of GPT-4o (OpenAI), accessed through the ChatGPT web interface (OpenAI, San Francisco, CA, USA; https://chatgpt.com).

The experiment consisted of 11 pairs comparing AI versus H and 11 pairs comparing HAI versus H. Each pair displayed two advertisements side-by-side, enabling direct visual comparison. For each trial, gaze metrics were first computed separately for each advertisement AOI, and were then aggregated per participant and condition as described in Section 3.6.

To minimize order effects and positional bias, both the sequence of stimulus presentation and the left–right positioning of advertisements were fully randomized for each participant. Furthermore, both advertisements in each pair referred to the same brand campaign, ensuring consistency in branding, product context, typography, and conceptual layout. The experimental design was intended to control major low-level visual properties across conditions. Mean luminance, RMS contrast, and computational saliency dispersion did not differ significantly between conditions. However, edge density was higher for AI-generated and HAI stimuli than for human-created stimuli. Consequently, creative origin and structural visual complexity were not fully separable in the present stimulus set, and the eye-tracking results are interpreted with this limitation in mind.

The two contrasts are not methodologically symmetric. Each HAI advertisement was derived directly from the human-created advertisement with which it was paired and therefore retained much of that advertisement’s layout, product depiction, and copy, whereas each AI-generated advertisement was produced independently from a text prompt. Prompt complexity also differed: AI-generated items were produced with detailed, item-specific creative prompts, whereas all HAI items were produced with the same generic refinement instruction. The HAI-versus-H contrast therefore compares two closely related versions of one design, while the AI-versus-H contrast compares two independently produced designs, and differences between the contrasts may reflect this asymmetry as well as the extent of AI involvement.

A comprehensive stimulus inventory detailing brand names, product categories, exact generative prompts, the refinement workflow, and image licenses is provided in Supplementary Material (Table S1). The exact prompt used for one AI-generated item (pair 11, Heinz) was not recorded; this item is documented only by its brand, model, and the product-focused nature of its prompt.

Participants were not informed that the advertisements differed in origin, and were not asked at any point to judge whether an advertisement was AI-generated. All results below therefore describe preference and gaze behavior under non-disclosure of stimulus origin. Because no source-identification task was administered after the experiment, it is not known whether participants spontaneously recognized some advertisements as AI-generated or AI-refined.

### 3.3. Participants

A total of N=39 participants took part in the study, aged between 18 and 26 years (M=20.35 years, SD=3.41). Gender distribution was not recorded during data collection. Participants were recruited via convenience and snowball sampling using direct word-of-mouth, WhatsApp messaging, and institutional email invitations. The sample included individuals from diverse educational backgrounds, ranging from secondary education to undergraduate studies, and consisted predominantly of undergraduate students.

All participants reported normal or corrected-to-normal vision, ensuring reliable visual perception during the experiment. Prior to participation, informed consent was obtained in accordance with ethical research standards. The study protocol was reviewed in accordance with the applicable institutional procedures at the Porto School of Engineering (ISEP). Formal institutional ethics committee review was waived because the study involved adult participants, non-invasive eye tracking, no intervention, and collection of anonymized behavioral and gaze data. All participants provided written informed consent before participation. No directly identifying personal information was retained with the experimental dataset.

Additionally, a preliminary questionnaire was administered to collect complementary profile information, such as familiarity with AI tools and digital media. A summary of the participants’ descriptive profiling is presented in Table 1, while the complete questionnaire instrument, verbatim item wording, and response scales are reported in Supplementary Material (Table S2). No a priori power analysis was conducted before data collection. A post hoc sensitivity analysis for a two-tailed paired comparison, based on the noncentral *t* distribution as implemented in G*Power 3 [31] (Heinrich Heine University Düsseldorf, Düsseldorf, Germany; https://www.psychologie.hhu.de/arbeitsgruppen/allgemeine-psychologie-und-arbeitspsychologie/gpower, accessed on 1 October 2026), indicated that, with 39 participants, α=0.05, and 80% power, the design could detect paired standardized effects of approximately $d_{z}\geq 0.46$. This analysis is presented as a sensitivity assessment rather than as evidence of adequate power for all hypotheses or outcome measures. Effects smaller than this threshold cannot be reliably distinguished from null, which is taken into account when interpreting non-significant ocular differences and which defines the equivalence bounds used in Section 3.6.

The study sample consisted of 39 participants, predominantly undergraduate students, reflecting a young and digitally immersed demographic. Social media engagement was substantial, with 79.5% reporting daily usage above two hours, underscoring the relevance of visually driven communication channels for this audience.

Marketing-related behaviors indicate moderate but meaningful engagement. A total of 38.5% expressed strong interest in marketing, and 43.6% acknowledged having made purchases influenced primarily by visual quality, highlighting the strategic importance of aesthetic cues in advertising targeted at younger consumers.

AI literacy emerged as a defining characteristic of the sample. A total of 92.3% reported frequent use of generative AI tools, and an equivalent proportion had previously engaged with AI-based image creation or editing. Despite this high familiarity, perceptions of AI-generated content remained ambivalent. While 51.3% believed AI could match human creativity, 56.4% simultaneously perceived AI-produced visuals as less authentic. This tension between technical appreciation and perceived authenticity is further reflected in ethical expectations: 69.2% of participants stated that companies should disclose AI usage in advertising materials.

Due to full anonymization of the profiling questionnaire, individual responses could not be deterministically linked to participant-level eye-tracking logs without risking matching errors. Consequently, these baseline attitudes serve as an aggregate sample-level characterization. Any comparison between these stated attitudes and the choices observed in the experiment (Section 5) therefore holds only at the group level: it describes the sample as a whole and cannot show that the participants who expressed a given attitude are the same participants who made a given choice. Overall, the descriptive profile reveals a digitally active, AI-experienced population with nuanced attitudes toward synthetic media. These baseline attitudes are of direct interest because they were measured before the task and can therefore be compared, at the group level, with the choices participants made when origin was not disclosed; this comparison is developed in Section 5.

### 3.4. Data Acquisition

Eye movement data were recorded using a Gazepoint GP3 eye-tracking system (Gazepoint Research Inc., Vancouver, BC, Canada) operating at a sampling rate of 60 Hz, controlled by Gazepoint Analysis UX Edition (v6.7.0, 64-bit) software for data acquisition and visualization. Stimuli were displayed on an integrated 15.6-inch flat-panel monitor with an active display area of 344.2 × 193.5 mm, at a spatial resolution of 1920×1080 pixels with a 60 Hz refresh rate. Participants were positioned at an approximate viewing distance of 65 cm, maintained using the software’s real-time optical position feedback indicator. At this distance, one screen pixel subtended approximately $0.0156^{\circ }$ of visual angle and the full display approximately $29.9^{\circ }\times 16.8^{\circ }$; this factor was used to express saccadic magnitudes and AOI dimensions in degrees of visual angle. Reporting of the recording set-up follows the minimal reporting guideline for eye-tracking studies [32] to the extent that the recorded information allows.

Before each experimental session, a standard 5-point calibration procedure was performed and validated to ensure accurate gaze tracking. During the experiment, participants were seated at a fixed distance from the display, and their gaze behavior was continuously recorded throughout stimulus presentation.

For all analyzed gaze metrics (fixation count, fixation duration, time to first fixation, saccadic magnitude, and pupil diameter), all N=39 participants provided valid data. Blink detection, however, was affected by partial loss of ocular reflection at 60 Hz: data from 5 participants exceeded the 20% track-loss threshold for continuous eyelid-closure detection. Because this would have reduced the sample for blink duration to n=34, and because blink events are only coarsely resolved at 60 Hz, blink duration was not retained as an inferential outcome in either contrast. All reported analyses are therefore based on the same N=39 participants and the same set of outcomes in both contrasts.

Fixations, saccades, and blinks were identified using the vendor default filtering algorithm embedded in Gazepoint Analysis UX Edition. Fixations were classified using the built-in Gazepoint spatial-temporal filter with a minimum fixation duration threshold of 45 ms (corresponding to approximately 3 consecutive samples at 60 Hz). Because the choice of event-detection algorithm and its parameters affects the resulting fixation and saccade measures [33,34], all recordings were processed with the same software version and settings. Blinks were identified as periods of pupil signal loss lasting between 15 ms (1 sample frame) and 2130 ms (2.13 s) bounded by valid tracking samples before and after the interruption. Signal losses exceeding this upper bound or lacking posterior validation were categorized as track loss rather than blinks; these criteria were used only to quantify track loss.

### 3.5. Procedure

The experimental sessions followed a structured, sequential protocol. Each session began with a standard 5-point calibration phase, followed by the presentation of 22 pairs of advertisements in randomized order. No practice trials were administered prior to the experimental task, ensuring that recorded gaze behavior reflected unprompted visual exploration and spontaneous decision-making from the first exposure. Pairs were presented in immediate succession, without an intervening central fixation cross or drift check, so gaze position at the onset of each pair was not controlled.

The viewing regime was self-paced: each stimulus pair remained on the screen until the participant responded by clicking on their preferred advertisement using the computer mouse. No artificial time limit was imposed, allowing for the capture of natural viewing behavior and unbiased attentional patterns under realistic decision-making conditions. Because trial presentation was fully participant-driven, no formal breaks were provided during the session. The entire experimental session was completed rapidly, lasting between 1 min 38 s and 4 min 30 s per participant (M=2min46s, SD=36.4 s).

### 3.6. Data Processing and Statistical Analysis

**Figure 2:**
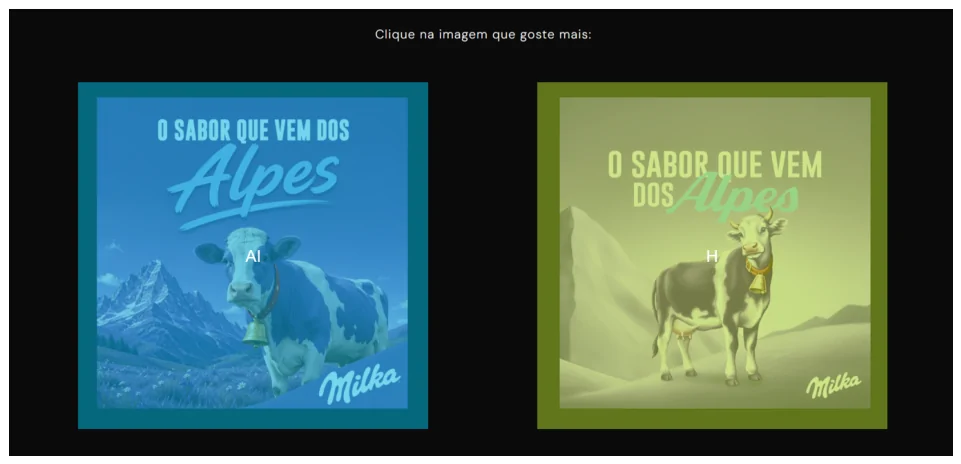
Representative stimulus pair illustrating the Areas of Interest (AOIs) corresponding to the full spatial extent of each advertisement condition. The condition labels (AI, H) were added for this illustration only and were not displayed to participants, who saw the two advertisements without any label. Each AOI coincides with the outer edges of the corresponding advertisement. Copyright of the advertisements remains with the respective brand owners; the images are reproduced solely for scientific illustration.

To evaluate attentional capture, visual exploration, and underlying cognitive processing, eye-tracking metrics were computed based on predefined Areas of Interest (AOIs). For each experimental pair, two primary rectangular AOIs were defined, corresponding to the full spatial extent of each individual advertisement (categorized by creative origin: Human, AI, or AI-refined), as illustrated in Figure 2. Each AOI coincided with the outer edges of the advertisement as displayed, and the two AOIs of a pair did not overlap. Standardized, fixed AOI dimensions were maintained across all stimulus presentation trials, measuring 900×740 px (≈$14.0^{\circ }\times 11.5^{\circ }$ of visual angle) with a fixed horizontal inter-AOI gap of 24 px ($\approx 0.37^{\circ }$). Sub-regions within each advertisement (brand, product, text) were not defined as separate AOIs, so the analyses describe the allocation of attention between the two advertisements and the scanning pattern within each advertisement as a whole, not the distribution of fixations over specific advertisement elements.

The data analysis phase incorporated two distinct categories of eye-tracking metrics to evaluate both attentional capture and the underlying cognitive processing:

**Visual Attention Metrics:**

- **Fixation Duration (FD):** The mean duration of individual fixations on the stimulus;
- **Fixation Count (FC):** The total number of individual fixations, summed over the 11 trials of the contrast, indicating the overall engagement and visual exploration density;
- **Time to First Fixation (TTFF):** The latency required for the participant’s gaze to enter a predefined Area of Interest (AOI), measuring initial attentional capture.

**Cognitive and Physiological Metrics:**

- **Pupil Diameter:** Pupil diameter was analyzed using the native units exported by the eye-tracking system, which are pixels of the eye-camera image and not screen pixels. Because no physical calibration or trial-level baseline correction was performed, these values cannot be converted to millimetres and were interpreted only as within-participant relative measures rather than as absolute physiological pupil diameter [35,36];
- **Saccadic Magnitude:** Saccadic magnitude was expressed in screen-space pixels. Because the same display configuration and viewing distance were used across conditions, the measure provides a consistent within-experiment spatial index; values are also given in degrees of visual angle, obtained with the pixel-to-degree factor reported in Section 3.4. Because a single factor is applied across the display, the conversion is approximate;
- **Blink Duration:** Recorded but not analyzed inferentially, for the reasons given in Section 3.4.

Statistical analyses were conducted in Python 3.12 (Python Software Foundation, Wilmington, DE, USA; https://www.python.org), using opencv-python (OpenCV; https://opencv.org) for image statistics and statsmodels (https://www.statsmodels.org) and SciPy (https://scipy.org) for inference; Microsoft Excel (Data Analysis ToolPak) and Microsoft Power BI (Microsoft Corporation, Redmond, WA, USA) were used for data management and visual exploration. The analysis plan for this study was not preregistered.

**Choice.** Each trial yields one binary outcome. For each contrast separately, choices were analyzed with an intercept-only binomial generalized linear model (logit link) with cluster-robust (sandwich) standard errors clustered by participant, which accounts for the repeated observations contributed by each participant. The intercept tests departure from chance (50%); its exponent is reported as an odds ratio (OR) with a 95% confidence interval, together with the log-odds estimate (β), its standard error, and the Wald *z* statistic. In both contrasts, the OR is the odds of choosing the alternative involving AI, that is, the AI-generated alternative in the AI-versus-H contrast and the AI-refined alternative in the HAI-versus-H contrast. To assess whether the two choice effects differ, the two log-odds estimates, both oriented toward the alternative involving AI, were compared with a Wald test of their difference; because the two estimates were treated as independent although they come from the same participants, this test is approximate.

**Response time.** Response time is a trial-level quantity shared by the two advertisements of a pair. It was analyzed with a linear mixed-effects model (statsmodels MixedLM, REML estimation) with the chosen alternative as fixed effect and a random intercept for participant; fixed effects are reported as β, standard error, and Wald *z*, together with the participant random-intercept variance.

**Eye-tracking measures.** Stimuli remained visible for a fixed 2.0 s post-response interval following the participant’s mouse click before transitioning to the next trial. Eye-tracking metrics were computed across the full trial presentation window (including the post-response display phase) to capture both pre-choice visual search and immediate post-decision inspection patterns. For each participant, the metrics of each AOI were aggregated over the 11 trials of each contrast (fixation count summed; fixation duration, time to first fixation, saccadic magnitude, and pupil diameter averaged), yielding one value per participant and condition. Conditions were compared with a one-way repeated-measures ANOVA with two levels, which is equivalent to a paired *t*-test ($F=t^{2}$, df=1,38). Effect sizes are reported as partial eta squared, $\eta_{p}^{2}=F/\left(F+df_{\mathrm{error}}\right)$, and 95% confidence intervals for the mean differences are based on the paired *t* distribution. Because this analysis is based on participant means, stimulus-level variance is not modeled and stimulus-level covariates such as edge density cannot be entered; inferences therefore refer to the present stimulus set. Because both AOIs of a trial share the same response time, response time is identical for the two levels of each comparison and cannot account for between-AOI differences. Fixation rate relative to fixation time (fixations per second fixated on an AOI) equals the reciprocal of mean fixation duration, so the fixation-duration test also serves as the test of fixation rate. Total fixation time (dwell time) on each AOI was computed at trial level as the summed duration of the fixations in that AOI, with a fixation still in progress at the response truncated at the response time, so that the summed fixation time on the two AOIs of a trial cannot exceed its response time. Dwell time was averaged per participant and condition over the 11 trials of each contrast and is reported descriptively.

**Equivalence.** For time to first fixation (H3), equivalence was assessed with two one-sided tests (TOSTs) [37], with bounds set to ±0.46 standard deviations of the paired differences, i.e., the smallest standardized effect the design could detect (Section 3.3), and with default Jeffreys–Zellner–Siow Bayes factors for paired designs (Cauchy prior scale r=0.707), reported as $BF_{01}$ in favor of the null hypothesis [38].

**Multiplicity.** Six prespecified eye-tracking outcomes (fixation duration, fixation count, time to first fixation, saccadic magnitude, and left- and right-eye pupil diameter) were evaluated for each of the two contrasts, resulting in 12 comparisons. Holm’s sequentially rejective procedure [39] was applied across this family to control the family-wise error rate, and only Holm-adjusted values ($p_{\mathrm{adj}}<0.05$) are interpreted as statistically significant.

## 4. Results

### 4.1. Log Analysis

A total of N=858 experimental trials were analyzed, consisting of forced-choice decisions between Human-created (H), AI-generated (AI), and AI-refined human (HAI) stimuli. The experimental design comprised two binary comparison paradigms comprising 429 trials each, with repeated observations within participants and stimulus pairs. The dependent variables were choice outcome and response time (ms).

**Figure 3:**
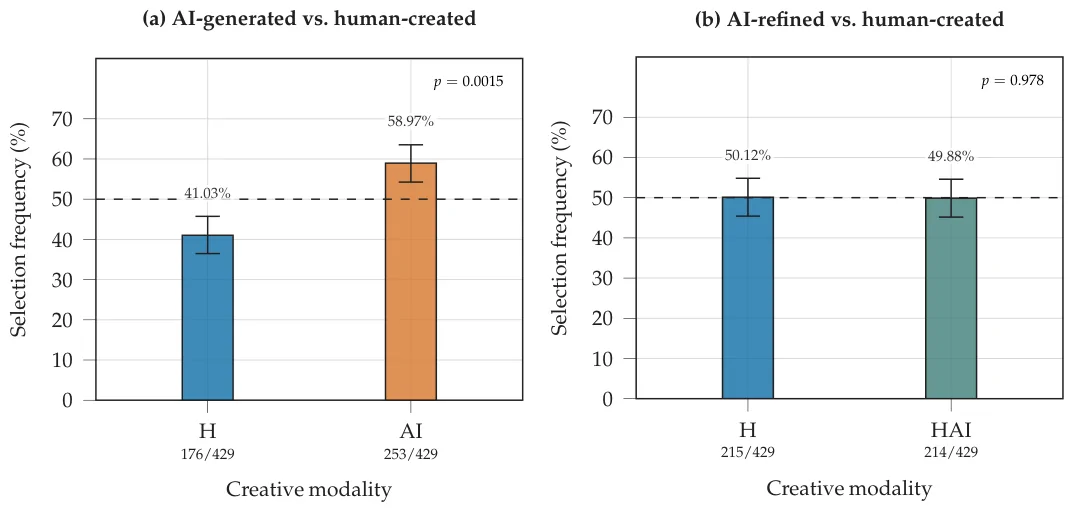
Selection frequency in the two forced-choice paradigms. Bars show the percentage of trials on which each alternative was chosen, with Wilson 95% confidence intervals (descriptive, not adjusted for clustering); the dashed line marks chance (50%). Trial counts are given beneath each bar (n=429 per contrast). The *p*-values are those of the binomial models with participant-clustered standard errors reported in Table 2 and Section 4.1.1.

Because response-time distributions are characteristically right-skewed, both parametric (Mean ± SD) and non-parametric (Median, IQR) statistics are reported to provide an accurate characterization of central tendency and dispersion. A comprehensive synthesis of these behavioral metrics is detailed in Table 2, with the overall preference distribution illustrated in Figure 3.

#### 4.1.1. Human vs. AI Choice Behavior

In the Human vs. AI condition, participants made strictly binary forced-choice decisions in which each trial required a direct comparison between a human-created and an AI-generated stimulus, with the outcome necessarily summing to 100% within each pair. Across all trials of this condition (n=429), human-created advertisements were selected in 41.03% of cases, whereas AI-generated advertisements were selected in 58.97% of cases, indicating an asymmetry in choice preference favoring AI-generated content.

To evaluate whether this deviation from an unbiased decision process (p=0.50 under the null hypothesis) was statistically reliable while accounting for trial-level dependencies within participants, an intercept-only binomial generalized linear model with standard errors clustered by participant was fitted (Section 3.6). The analysis revealed that the observed choice distribution differed significantly from chance expectation (β=0.363, SE=0.114, z=3.17, p=0.0015).

The magnitude of this effect was quantified using an odds ratio, which indicated that the odds of selecting the AI-generated advertisement were 1.44 times the odds of selecting the human-created advertisement (OR=1.44). The associated 95% confidence interval [1.15,1.80] lies entirely above unity, indicating a preference for AI-generated over human-created advertisements under non-disclosure of origin, in this sample and stimulus set.

#### 4.1.2. Human vs. HAI Choice Behavior

In the Human vs. HAI condition, participants again made strictly binary forced-choice decisions, with each trial requiring a direct comparison between a human-created and an AI-refined stimulus, such that selections summed to 100% within pairs. Across all trials of this contrast (n=429), human-created advertisements were chosen in 50.12% of trials, whereas AI-refined advertisements were selected in 49.88% of trials.

The same model was fitted to assess whether this deviation from the null hypothesis of equal choice probability (p=0.50) was statistically significant. The results showed no statistically significant departure from chance expectation (β=−0.005, SE=0.171, z=−0.03, OR=0.99, 95%CI[0.71,1.39], p=0.978; odds of choosing the AI-refined alternative).

These results provide no evidence of a preference in either direction between human-created and AI-refined advertisements. Because the 95% confidence interval includes unity and spans odds ratios between roughly 0.71 and 1.39, this non-significant result represents a failure to reject the null hypothesis rather than proof of population equivalence.

#### 4.1.3. Effect Size Comparison and Response Time Analysis

The two contrasts differ descriptively in outcome: AI-generated advertisements were preferred over human-created ones (OR=1.44, 95%CI[1.15,1.80]), whereas the human-versus-AI-refined contrast was indistinguishable from chance (OR=0.99, 95%CI[0.71,1.39]). To test whether the two effects differ, the log-odds of choosing the alternative involving AI (${\mathrm{OR}}_{\mathrm{AI}}=1.44$; ${\mathrm{OR}}_{\mathrm{HAI}}=0.99$) were compared. The ratio of the two odds ratios was 1.44 (95% CI [0.96,2.16]; z=1.78, p=0.074). The difference between the two choice effects is therefore not statistically reliable in the present data, and this test is approximate because the two estimates come from the same participants. Therefore, no graded or monotonic relationship between the degree of AI involvement and preference is claimed from these data, and the two contrasts are not described as differing in preference.

Response times were analyzed using a linear mixed-effects model with the chosen alternative as fixed effect and a random intercept for participant. The analysis showed that response times did not differ significantly between trials on which each alternative was chosen, in either contrast. For the Human vs. AI contrast, the effect was not significant (β=126.92, SE=257.15, z=0.49, p=0.622; participant random-intercept variance $=3.17\times 10^{6}$). Similarly, for the Human vs. HAI contrast, no significant difference was observed (β=435.39, SE=280.27, z=1.55, p=0.120; participant random-intercept variance $=2.97\times 10^{6}$).

Descriptively, in the Human vs. AI contrast, ${\mathrm{RT}}_{\mathrm{Human}}=5658.9\pm 3101.9$ ms and ${\mathrm{RT}}_{\mathrm{AI}}=5524.6\pm 2992.0$ ms; in the Human vs. HAI contrast, ${\mathrm{RT}}_{\mathrm{Human}}=5216.8\pm 2868.8$ ms and ${\mathrm{RT}}_{\mathrm{HAI}}=5212.6\pm 3206.2$ ms. The apparent difference in overall response time between the two experimental blocks (approximately 5.6 s vs. 5.2 s) was not subjected to a formal between-block statistical test and is therefore not interpreted as a meaningful effect.

### 4.2. Eye-Tracking Analysis

Following the methodological framework described above, this section presents the empirical results obtained from the eye-tracking experiment. The analysis quantifies differences in visual attention across human-created, AI-generated, and AI-refined advertisements using the metrics defined in Section 3.6: Fixation Duration (FD), Fixation Count (FC), Time to First Fixation (TTFF), Saccadic Magnitude, and Pupil Diameter.

All comparisons were within-subject repeated-measures analyses of participant-level means (N=39). Six outcome measures were tested per contrast: fixation duration, fixation count, time to first fixation, saccadic magnitude, left-eye pupil diameter, and right-eye pupil diameter, giving 12 comparisons. Uncorrected *p*-values are reported for completeness, but inference is based on Holm-adjusted values [39], which are given in Table 3 and Table 4. Partial eta squared is reported as an effect size for each test.

#### 4.2.1. AI-Generated vs. Human-Created

**Figure 4:**
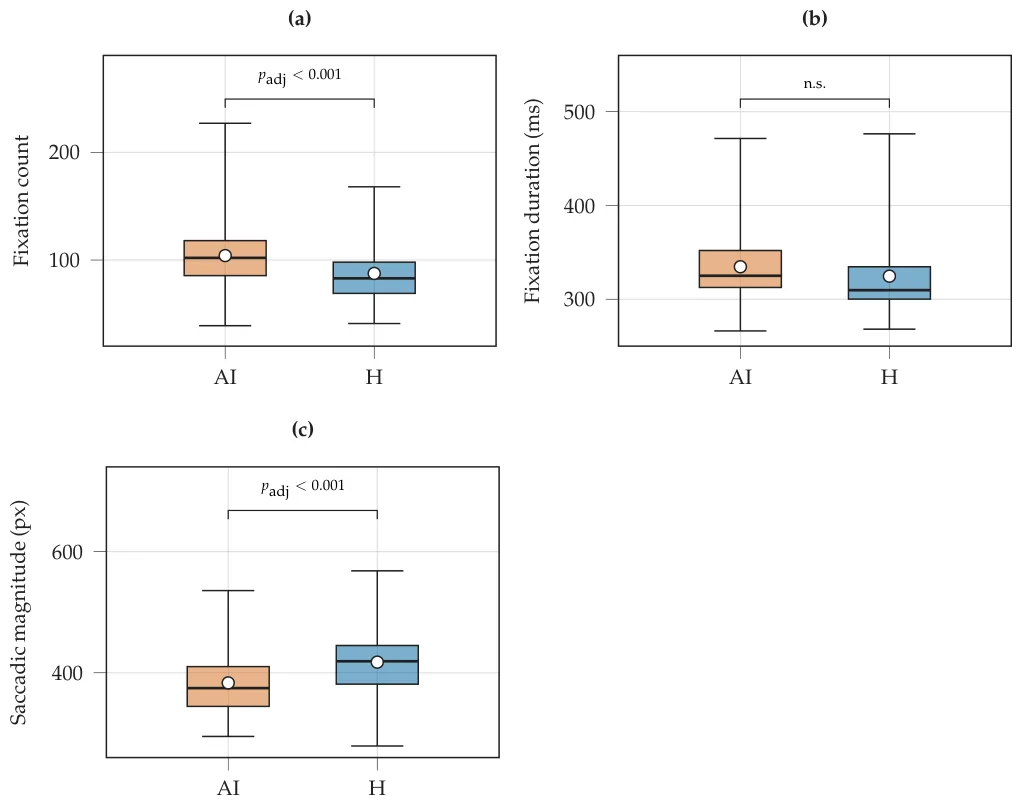
Distributions of the eye-tracking metrics for AI-generated (orange) and human-created (blue) advertisements: (**a**) fixation count, (**b**) fixation duration, (**c**) saccadic magnitude. Boxes span the interquartile range, the horizontal rule is the median, the white circle is the mean, and whiskers extend to the minimum and maximum. Brackets give the Holm-adjusted *p*-value from the repeated-measures ANOVA on participant-level means (Table 3); n.s. denotes a comparison that does not survive correction. N=39 participants. Axis ranges are shared with the corresponding panels of Figure 5.

This section compares the visual attention patterns elicited by AI-generated and human-created advertisements. Table 3 presents descriptive statistics, mean differences with 95% confidence intervals, inferential results, and effect sizes for the two conditions. The corresponding distributions are shown in Figure 4.

**Figure 5:**
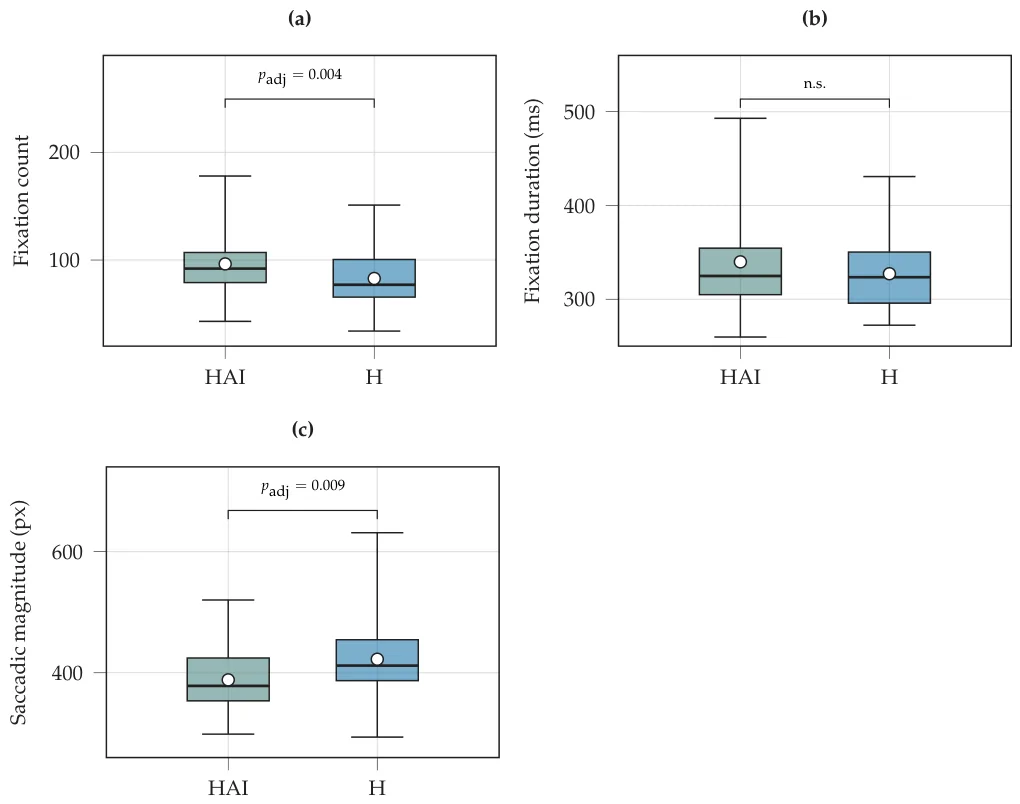
Distributions of the eye-tracking metrics for AI-refined HAI (teal) and human-created (blue) advertisements: (**a**) fixation count, (**b**) fixation duration, (**c**) saccadic magnitude. Conventions and axis ranges are identical to Figure 4. Brackets give the Holm-adjusted *p*-value from Table 4. N=39 participants.

Two effects survive correction for multiple comparisons. AI-generated advertisements elicited a higher fixation count than human-created advertisements (104.13±32.66 vs. 87.56±26.50; meandifference=16.57, F(1,38)=34.26, $p_{\mathrm{adj}}<0.001$, $\eta_{p}^{2}=0.47$) and smaller saccadic magnitudes (383.36±52.80 vs. 417.63±61.93 px; meandifference=−34.27 px, about $-0.55^{\circ }$, F(1,38)=35.18, $p_{\mathrm{adj}}<0.001$, $\eta_{p}^{2}=0.48$). Together, these indicate that AI-generated advertisements received more fixations, connected by shorter saccades, than their human-created counterparts, consistent with H1 and H2 at the level of the stimulus sets.

No other comparison survived correction. The difference in fixation duration was not statistically significant before correction and not significant after it (F(1,38)=4.02, p=0.052, $p_{\mathrm{adj}}=0.365$, $\eta_{p}^{2}=0.10$). Time to first fixation did not differ between conditions (F(1,38)=0.07, p=0.792, $p_{\mathrm{adj}}=1.000$, $\eta_{p}^{2}<0.01$). The TOST procedure with bounds of ±283 ms (±0.46 SD of the paired differences) indicated statistical equivalence (p=0.006), and the Bayes factor favored the null hypothesis ($BF_{01}=5.6$), consistent with H3. Pupil diameter did not differ for either eye (both p>0.18).

Because the fixation-duration difference was small and non-significant, fixation rate relative to fixation time (the reciprocal of mean fixation duration; approximately 2.99 vs. 3.08 fixations per second) did not differ between conditions, and the higher fixation count therefore does not reflect denser sampling. Instead, it was accompanied by more total fixation time on the AI-generated advertisement: computed at trial level, mean dwell time per trial was 3.16s on the AI-generated and 2.58s on the human-created advertisement, or about 55.05% of the fixation time within a pair, against a mean response time of 5.58 s in this contrast.

#### 4.2.2. AI-Refined vs. Human-Created

This section examines the differences in visual attention between AI-refined human (HAI) and human-created advertisements. Table 4 presents descriptive statistics, mean differences with 95% confidence intervals, inferential results, and effect sizes for the two conditions, and Figure 5 shows the corresponding distributions.

As in the previous contrast, two effects survive Holm correction. HAI advertisements elicited a higher fixation count than human-created advertisements (96.38±29.15 vs. 82.87±27.37; meandifference=13.51, F(1,38)=15.39, $p_{\mathrm{adj}}=0.004$, $\eta_{p}^{2}=0.29$) and smaller saccadic magnitudes (388.36±49.81 vs. 422.44±63.99 px; meandifference=−34.08 px, about $-0.55^{\circ }$, F(1,38)=12.84, $p_{\mathrm{adj}}=0.009$, $\eta_{p}^{2}=0.25$). The direction and approximate magnitude of both effects are similar to those observed in the AI-vs.-H contrast, consistent with H1 and H2 for the AI-refined stimulus set.

The difference in fixation duration (F(1,38)=4.91, p=0.033, $p_{\mathrm{adj}}=0.262$, $\eta_{p}^{2}=0.11$) did not survive correction and is reported as exploratory. Time to first fixation was effectively identical between conditions (732.26±337.97 vs. 732.13±404.93 ms; p=0.998; TOST with bounds of ±170 ms, p=0.003; $BF_{01}=5.8$), again consistent with H3, and pupil diameter did not differ for either eye (both p>0.67). As in the first contrast, fixation rate relative to fixation time did not differ (approximately 2.94 vs. 3.06 fixations per second), while total fixation time was higher for the AI-refined advertisement (computed at trial level, 2.97s vs. 2.48s per trial, or about 54.50% of the fixation time within a pair, against a mean response time of 5.21 s in this contrast).

## 5. Discussion

Two main findings require explanation. First, under non-disclosure of origin, AI-generated advertisements were chosen significantly more often than human-created ones (OR=1.44, p=0.0015), whereas AI-refined advertisements were chosen no more and no less often than human-created ones (OR=0.99, 95% CI [0.71,1.39], p=0.978). Second, both AI-generated and AI-refined advertisements were associated with a similar gaze pattern relative to human-created advertisements—a higher fixation count and smaller saccadic magnitudes—with medium-to-large effect sizes in both contrasts. These two findings do not align directly: the gaze pattern shared by AI and HAI stimuli was not accompanied by the same choice outcome. This divergence is descriptive, however, because the difference between the two choice effects was not statistically reliable (p=0.074).

A parsimonious interpretation of the gaze findings is that structural and compositional properties contributed substantially to the observed pattern.

Two aspects of the gaze pattern need to be distinguished. The higher fixation count was not accompanied by a higher fixation rate relative to fixation time; it reflects a larger share of fixation time directed to the AI-generated or AI-refined advertisement (about 55.05% and 54.5% in the two contrasts). The smaller saccadic magnitudes, in contrast, describe how gaze moved within and between the advertisements, with shorter transitions for the AI-involved stimulus sets. Analysis of low-level visual properties showed that while stimuli did not differ significantly in mean luminance (p=0.861), RMS contrast (p=0.531), and computational saliency dispersion (p=0.115), synthetic and AI-refined stimuli exhibited higher structural complexity (edge density: $M_{\mathrm{AI}}=59.09$ and $M_{\mathrm{HAI}}=61.56$ vs. $M_{H}=40.73$; Section 3.1). Generated imagery from current diffusion models tends toward highly detailed, visually concentrated focal elements, whereas human-designed advertisements more broadly distribute informative components—copy, logo, product, call to action—across the canvas. This elevated edge density and spatial concentration of visual detail in AI/HAI imagery offers a plausible stimulus-driven explanation for both longer viewing and shorter saccades. Because complexity differences remain present, this visual effect cannot be disentangled from a genuine processing difference with the data at hand [18]. The data therefore do not support the stronger claim that synthetic imagery is intrinsically more efficient to process.

The equivalence of time to first fixation across all conditions is informative in this respect. If AI-generated stimuli possessed a sizeable saliency advantage in capturing initial attention, a systematic difference in visual capture would be expected; none was observed in either contrast, with the AI-refined and human means differing by a mere 0.13 ms, and equivalence tests and Bayes factors supported the absence of effects of the size the design could detect. Two caveats apply. Effects smaller than about 0.46 SD cannot be excluded, and gaze position at pair onset was not controlled by a fixation cross, so TTFF partly reflects where gaze happened to be when each pair appeared. Because left–right placement was randomized, this starting-position variability is balanced across conditions in expectation, but it adds noise to the measure. Whatever distinguishes visual processing across conditions appears to emerge during ongoing exploration rather than at onset.

The preference result should be interpreted with three constraints firmly in view. The first is disclosure. Participants were never told that stimulus origin varied and were never asked to judge it, so the observed preference for AI-generated advertisements was formed in the absence of authorship information. The literature on anthropocentric bias indicates that appreciation of visual work falls when machine authorship is revealed [9,10], and that algorithmic provenance depresses evaluation across domains [11,12,13]. The present sample makes this constraint concrete: 56.4% of these same participants reported that AI-generated visuals are less authentic, and 69.2% reported support for mandatory disclosure, while AI-generated advertisements were selected in 58.97% of trials. Because the questionnaire was fully anonymous, this comparison holds only at the group level: it cannot establish that the participants who endorsed these attitudes are the ones who chose the AI-generated advertisements.

This is a divergence between stated attitude and revealed choice at the level of the sample, not evidence that the stated attitude is inert. A disclosure manipulation is the natural next experiment, and until it is run, the practical implication of the present result is limited to contexts in which origin is not disclosed—which is precisely the context that the majority of these participants said should not exist.

The second constraint is the coupling between gaze and choice inherent in the paradigm. In binary preference tasks, gaze shifts progressively toward the alternative that is eventually chosen [19], and dwell time and choice are jointly determined during evidence accumulation [20]. Because the two advertisements in each pair were presented simultaneously and competed for a bounded viewing period, fixation measures on the two sides are not independent. The reported fixation-count difference in the AI-vs.-H contrast is therefore partly redundant with the choice difference in that same contrast. Some evidence against this concern comes from the HAI-vs.-H contrast, where the same gaze pattern appears in the absence of any choice asymmetry: AI-refined stimuli drew more fixations and shorter saccades while being chosen about as often as human-created ones. The pattern is consistent with H4, in that the similarity of the gaze effects across AI and HAI contrasts was not accompanied by equivalent choice outcomes, although the difference between the two choice effects was not itself statistically significant.

The third constraint is the asymmetry between the two contrasts. Each AI-refined advertisement was derived from the human-created advertisement it was paired with and shared much of its content, whereas each AI-generated advertisement was produced independently from a detailed prompt. Two near-identical versions of one design may be harder to discriminate, which could by itself move choices toward chance in the HAI-versus-H contrast; conversely, independently generated AI advertisements could differ from their human counterparts in many respects besides origin. The chance-level result for AI-refined advertisements therefore cannot be read as evidence about human–AI co-creation in general, which in professional practice usually involves iterative human direction that was absent here.

It is worth stating explicitly which theoretical prediction the AI-refined result fails to support. A category-ambiguity account of the uncanny valley predicts that stimuli of indeterminate origin are evaluated more negatively than stimuli of unambiguous origin, as difficulty of categorization is itself aversive [40,41]. Applied to advertising, this predicts that material of mixed human and AI origin should be penalized relative to both fully human and fully synthetic material. No such penalty was observed: AI-refined advertisements were chosen at chance level (49.88%; OR=0.99, 95% CI [0.71,1.39]). A processing-fluency account [42], on which more easily processed stimuli are preferred, fares no better here: AI-refined and AI-generated stimuli produced the same gaze pattern, yet only the latter was preferred. The present design does not provide a strong test of either framework.

Blink duration was not analyzed inferentially because of track loss in five participants and the limited temporal resolution of 60-Hz recording, and no independent measure of cognitive workload was collected against which blink measures could have been validated [43]. The pupil results are similarly uninformative as reported: although mean luminance did not differ significantly across experimental conditions (p=0.861), the absence of trial-level pre-stimulus baseline correction limits the evidential weight of a null pupil effect in indexing subtle cognitive load differences [35,36].

Taken together, the results are most consistent with the view that generative and AI-refinement pipelines currently produce advertisements whose informative content is spatially concentrated and structurally dense (higher edge density), and that this compositional property is associated with how the image is scanned without determining whether it is preferred. For practitioners, the actionable implication is narrow but real: AI-assisted production did not degrade attentional engagement or choice relative to human production in this stimulus set, and AI refinement of human advertisements in particular achieved parity on choice. The evidence does not support recommendations about “optimizing” cognitive load, which the present measures cannot speak to. The elevated edge density of the AI-generated and HAI stimuli is therefore not merely a secondary descriptive difference but a potential alternative explanation for the observed gaze pattern. Higher edge density may increase the number of visually informative local features and consequently alter fixation placement and saccadic transitions. Because edge density was not experimentally controlled, and could not be entered as a covariate in analyses based on participant-level means, the data do not allow us to determine whether the observed differences are attributable to creative origin, structural complexity, or their combination. Future experiments should therefore construct stimuli in which edge density and other low-level visual properties are matched across origin conditions, and should analyze trial-level data with crossed random effects for participants and stimuli [44] and with stimulus-level covariates.

Finally, all findings refer to a specific population: young adults aged 18–26 years, predominantly undergraduate students, of whom 92.3% were frequent users of generative-AI tools. Such a sample may be more familiar with, and possibly more tolerant of, AI-generated imagery than the general consumer population, and the conclusions are restricted accordingly until they are replicated with older, less AI-experienced, and more diverse samples.

## 6. Limitations

Several limitations constrain the interpretation of these results.

**Sample.** The 39 participants were predominantly undergraduate students from a single institution, with a mean age of 20.35 years (SD=3.41; range 18–26) and an unusually high level of generative-AI experience (92.3% frequent users). Gender was not recorded. This is not a consumer sample, and the results, and the preference results in particular, apply to young adults with high AI literacy and should not be generalized to broader populations without replication. No a priori power analysis was conducted.

**Stimuli.** Although stimuli across experimental conditions did not differ significantly in mean luminance, RMS contrast, and computational saliency dispersion, synthetic and AI-refined stimuli exhibited higher structural complexity (edge density) than human-created controls. Consequently, a stimulus-driven, compositional explanation for the observed gaze pattern cannot be fully disentangled from an intrinsic processing difference with the current visual corpus [18]. The AI-refined condition was produced with a single generic refinement prompt applied to the paired human advertisement, whereas AI-generated items were produced independently with detailed prompts; the two contrasts are therefore not symmetric, and differences in prompt complexity may have contributed to differences in image quality. The prompt for one AI-generated item was not recorded. The advertisements contained Portuguese text, and the accuracy of the text rendered by the generative model was not formally assessed; distorted typography is therefore a possible additional source of differences between conditions. Only 11 items per contrast were used. The human-created advertisements are published brand material, and their use was limited to research purposes.

**Design.** Origin was never disclosed to participants and never explicitly queried, meaning the study cannot determine whether participants implicitly recognized AI-generated imagery, nor how visual preference would behave under formal authorship disclosure. No post-experiment source-identification task was administered. No fixation cross or drift check preceded each pair, so initial gaze position was not controlled, which particularly affects time to first fixation. Because the profiling questionnaire was anonymous, attitudes and choices can be related only at the group level.

**Measurement.** The 60 Hz sampling rate of the eye tracker limits the temporal resolution of fast oculomotor events such as blinks and short saccades; for this reason, and because of track loss in five participants, blink duration was not analyzed. Saccadic magnitude was recorded in screen pixels and converted to degrees of visual angle with a single pixel-to-degree factor, which is approximate, and pupil diameter was recorded in eye-camera pixels and could not be converted to millimetres. Additionally, pupil size metrics lacked trial-level pre-stimulus baseline correction, which limits the sensitivity of pupil data in indexing fine-grained cognitive workload differences [35,36]. AOIs covered whole advertisements, so the distribution of fixations over brand, product, and text regions was not examined.

**Analysis.** The statistical analysis plan was not preregistered, and multiple eye-tracking outcomes were evaluated across two contrasts. The hypotheses were also finalized after the data had been collected and analyzed, so their tests are not tests of predictions fixed in advance. Although Holm correction was applied across the family of 12 comparisons, the possibility of analytical flexibility cannot be excluded. Eye-tracking measures were analyzed as participant-level means, and choices with participant-clustered standard errors; neither analysis models variance between stimuli, so the results may not generalize beyond the present stimulus set, and edge density could not be included as a covariate. Dwell time was reported descriptively and was not tested, and the comparison of the two choice effects is an approximate test. Future studies should preregister primary outcomes, model specifications, exclusion criteria, and multiplicity procedures before data collection.

## 7. Conclusions

This study compared human-created, AI-generated, and AI-refined human advertisements using eye-tracking and a forced-choice preference task with 39 young, AI-experienced participants and 858 trials, under non-disclosure of stimulus origin.

Two eye-tracking effects survived correction for multiple comparisons in both contrasts: relative to human-created advertisements, AI-generated and AI-refined advertisements were associated with more fixations ($\eta_{p}^{2}=0.47$ and 0.29) and smaller saccadic magnitudes ($\eta_{p}^{2}=0.48$ and 0.25). The additional fixations reflected a larger share of fixation time on these advertisements rather than a higher fixation rate. Initial attentional capture, indexed by time to first fixation, was equivalent across all conditions within the bounds the design could detect, and pupil diameter did not differ. Differences in fixation duration did not survive correction and are not interpreted.

Behaviorally, AI-generated advertisements were selected more often than human-created ones (OR =1.44, 95% CI [1.15,1.80], p=0.0015), whereas the human-versus-AI-refined comparison was indistinguishable from chance (OR =0.99 for choosing the AI-refined alternative, 95% CI [0.71,1.39], p=0.978). Response times did not differ within either contrast.

The central empirical observation of this work is that AI-generated and AI-refined advertisements shared a similar gaze pattern (high fixation count, short saccades), while only AI-generated advertisements were preferred over human-created ones. Because the difference between the two choice effects was not statistically reliable, this is a descriptive divergence rather than an established dissociation; it nonetheless indicates that the gaze pattern observed here is not, by itself, a sufficient predictor of choice. Three qualifications bound this conclusion. First, the preference for AI imagery was observed in the absence of authorship disclosure, in a sample in which 69.2% of participants, at the group level, expected disclosure in commercial contexts. Second, while stimuli did not differ significantly in mean luminance, RMS contrast, and computational saliency, synthetic and AI-refined imagery exhibited elevated structural complexity (edge density), so the observed gaze pattern may reflect the spatial composition and detail density typical of current generative pipelines rather than creative origin as such. Third, the AI-refined condition was derived from its paired human advertisement with a generic prompt, and the sample consisted of young adults with high AI literacy.

Several directions for future research emerge. First, a formal disclosure manipulation is required to evaluate whether the preference for AI-generated advertisements persists when algorithmic provenance is made explicit, complemented by a post-experiment source-identification task. Second, isolating specific structural parameters (e.g., controlling edge density alongside saliency) will further separate spatial-compositional effects from cognitive processing mechanisms. Third, trial-level models with crossed random effects for participants and stimuli, stimulus-level covariates, and a formal contrast-by-condition interaction will provide a direct test of the attention–preference coupling in generative visual media. Fourth, analyses of sub-regions (brand, product, text), an assessment of AI-rendered typography, human–AI workflows with genuine iterative human direction, and more diverse samples are needed to establish how far the present findings generalize.

## Figures and Tables

**Table 1:** Descriptive profile and behavioral characterization of the study sample (N=39). Items measured on a 5-point Likert scale (1 = Strongly Disagree to 5 = Strongly Agree) were dichotomized by reporting the proportion of participants selecting top-box responses (4 = Agree or 5 = Strongly Agree), except where specific behavioral criteria are noted.

Dimension	Metric/Behavioral Indicator	n/N	(%)
Digital Engagement	Social media usage (>2 h/day)	31/39	79.5%
Marketing	Strong interest in marketing ^1^	15/39	38.5%
	Purchases influenced by visual quality	17/39	43.6%
AI Experience	Frequent use of generative AI tools	36/39	92.3%
	Prior engagement with AI image creation/editing	36/39	92.3%
AI Perceptions	Belief that AI matches human creativity ^1^	20/39	51.3%
	Perception of AI visuals as less authentic ^1^	22/39	56.4%
	Expectation of mandatory AI disclosure by brands ^1^	27/39	69.2%

^1^ Indicates items originally measured on a 5-point Likert scale, reported as the percentage of top-box respondents (4 or 5).

**Table 2:** Behavioral results synthesis for the forced-choice decision tasks (N=858 total trials; n=429 per contrast). Odds ratios, confidence intervals, and *p*-values are from intercept-only binomial models with participant-clustered standard errors. In both contrasts, the OR is the odds of choosing the alternative involving AI (AI-generated or AI-refined); in the Human vs. HAI contrast, the equivalent odds of choosing the human-created alternative are OR=1.01, 95% CI [0.72,1.41]. Response times are those of the trials on which the corresponding alternative was chosen.

Metric/Variable	Human vs. AI		Human vs. HAI	
	Human	AI	Human	AI-Refined (HAI)
Choice Preference (%)	41.03%	58.97%	50.12%	49.88%
Odds Ratio (OR)	—	1.44	—	0.99
95% Confidence Interval	—	[1.15, 1.80]	—	[0.71, 1.39]
*p*-value	0.0015		0.978	
Response Time (ms)				
Mean ± SD	5658.9 ± 3101.9	5524.6 ± 2992.0	5216.8 ± 2868.8	5212.6 ± 3206.2
Median	4959.5	4806	4458	4467
IQR ($Q_{1}$–$Q_{3}$)	4050.25	3318.5	3115	3362.25

**Table 3:** Comparison of eye-tracking metrics between AI-generated and human-created advertisements. Values are mean ± SD of participant-level values (N=39); *F*, *p*, and $\eta_{p}^{2}$ are from repeated-measures ANOVAs. $p_{\mathrm{adj}}$ is the Holm-adjusted *p*-value across all 12 tests reported in Section 4.2. FC is summed over the 11 trials of the contrast; pupil values are in eye-camera pixels.

Metric	AI-Generated	Human-Created	Mean Difference [95% CI]	*F*	df	*p*	$p_{\mathrm{adj}}$	$\eta_{p}^{2}$
FD (ms)	334.69 ± 41.21	324.66 ± 43.90	10.03 [−0.10, 20.15]	4.02	1, 38	0.052	0.365	0.096
FC	104.13 ± 32.66	87.56 ± 26.50	16.57 [10.84, 22.30]	34.26	1, 38	<0.001	<0.001	0.474
TTFF (ms)	795.98 ± 588.38	769.89 ± 403.17	26.09 [−173.5, 225.7]	0.07	1, 38	0.792	1.000	0.002
Saccade (px)	383.36 ± 52.80	417.63 ± 61.93	−34.27 [−45.97, −22.57]	35.18	1, 38	<0.001	<0.001	0.481
Pupil left (px)	23.22 ± 4.29	23.32 ± 4.31	−0.10 [−0.25, 0.05]	1.82	1, 38	0.185	1.000	0.046
Pupil right (px)	22.91 ± 4.46	22.89 ± 4.57	0.02 [−0.18, 0.22]	0.04	1, 38	0.842	1.000	0.001

**Table 4:** Comparison of eye-tracking metrics between AI-refined and human-created advertisements. Values are mean ± SD of participant-level values (N=39); *F*, *p*, and $\eta_{p}^{2}$ are from repeated-measures ANOVAs. $p_{\mathrm{adj}}$ is the Holm-adjusted *p*-value across all 12 tests reported in Section 4.2. FC is summed over the 11 trials of the contrast; pupil values are in eye-camera pixels.

Metric	AI-Refined	Human-Created	Mean Difference [95% CI]	*F*	df	*p*	$p_{\mathrm{adj}}$	$\eta_{p}^{2}$
FD (ms)	339.92 ± 52.24	327.25 ± 38.14	12.67 [1.09, 24.25]	4.91	1, 38	0.033	0.262	0.114
FC	96.38 ± 29.15	82.87 ± 27.37	13.51 [6.54, 20.48]	15.39	1, 38	<0.001	0.004	0.288
TTFF (ms)	732.26 ± 337.97	732.13 ± 404.93	0.13 [−119.5, 119.7]	<0.01	1, 38	0.998	1.000	<0.001
Saccade (px)	388.36 ± 49.81	422.44 ± 63.99	−34.08 [−53.33, −14.83]	12.84	1, 38	0.001	0.009	0.253
Pupil left (px)	23.10 ± 4.46	23.10 ± 4.32	0.00 [−0.14, 0.14]	0.004	1, 38	0.951	1.000	<0.001
Pupil right (px)	22.83 ± 4.70	22.80 ± 4.63	0.03 [−0.11, 0.17]	0.18	1, 38	0.675	1.000	0.005

## Data Availability

The anonymized participant-level and trial-level datasets, the image-statistics and analysis scripts, and the full stimulus inventory are available on reasonable request to the authors.

## Supplementary Materials

The following supporting information can be downloaded at https://www.mdpi.com/article/10.3390/s26206411/s1. Table S1: Full stimulus inventory across the 22 experimental advertisement pairs (11 AI vs. H and 11 HAI vs. H; HAI = AI-refined human advertisement). All prompts were executed in Portuguese and translated into English for reporting. All images were generated with the native image-generation capability of GPT-4o (OpenAI) in the ChatGPT interface, denoted GPT-4o below. For HAI items, original human advertisements (sourced from official brand campaigns on Google Images and Instagram) were uploaded with a standardized refinement prompt, allowing the model to autonomously refine visual appeal; no further human guidance was given, so this condition represents AI refinement of a human design. Human-created advertisements were obtained from publicly accessible official brand sources and used solely as experimental stimuli. Copyright ownership remains with the respective rights holders. Table S2: Complete inventory of verbatim questionnaire items, constructs, and response scales (administered in Portuguese).
