# Supplementary material for: Eye-Tracking Analysis of Visual Attention and Preference in Human-Created, AI-Generated, and AI-Refined Human Advertisements
Source: Sensors (Basel). 2026 Oct 10;26(20):6411. doi: 10.3390/s26206411 (PMC13709040; doi:10.3390/s26206411)
Supplement: Supplementary file 1 [file sensors-26-06411-s001.zip › sensors-4569970-supplementary.pdf]

Supplementary Materials

Eye-Tracking Analysis of Visual Attention and Preference in Human-Created, AI-Generated, and AI-Refined Human Advertisements

André Gonçalves, Margarida Afonso, Pedro Martins and Ana Teixeira

Supplementary Materials: Stimulus Inventory

Table S1: Full stimulus inventory across the 22 experimental advertisement pairs (11 AI vs. H and 11 HAI vs. H; HAI = AI-refined human advertisement). All prompts were executed in Portuguese and translated into English for reporting. All images were generated with the native image-generation capability of GPT-4o (OpenAI) in the ChatGPT interface, denoted GPT-4o below. For HAI items, original human advertisements (sourced from official brand campaigns on Google Images and Instagram) were uploaded with a standardized refinement prompt, allowing the model to autonomously refine visual appeal; no further human guidance was given, so this condition represents AI refinement of a human design. Human-created advertisements were obtained from publicly accessible official brand sources and used solely as experimental stimuli. Copyright ownership remains with the respective rights holders.

Pair	Contrast	Brand	AI Model & Exact Prompt
1	AI vs. H	Compal	GPT-4o: “Create a 3x4 advertising poster for the Compal brand, with a background of strawberries and cherries, in the center a Compal bottle with the new strawberry and cherry flavor, on the bottle label it must have the brand Compal 1952 Vital Proteção Anti-OX, strawberry cherry, on the poster it must say ’A cereja no topo do VERÃO’ with emphasis on the word ’VERÃO’.”
2	AI vs. H	Gillette	GPT-4o: “Create a 1080x1080 advertising poster for the Gillette brand, with a blue background, in the center it must say ’MOSTRA A TUA IDENTIDADE’ in capital letters, the brand centered at the top, a white razor in the upper left corner, and a black razor in the lower right corner.”
3	AI vs. H	Olá	GPT-4o: “Create a 1024x1024 advertising poster for the Olá brand, with a purple background, in the center featuring César Mourão, Portuguese actor and comedian, eating a Magnum Almond ice cream, in the upper left corner the Olá heart-shaped logo with a red background, in the upper right corner written in uppercase ’COME À GRANDE’, and in the lower left corner written in uppercase ’A FELICIDADE NÃO TEM REGRAS’.”
4	AI vs. H	Dodot	GPT-4o: “Create a 1024x1024 advertising poster for the Dodot brand, with a blue background with transparent stars, on the left side a baby lying down sleeping only in a diaper, on the left side written ’Tão Tão’, below that the Dodot brand, and below that written ’bebé seco’.”
5	AI vs. H	Donuts	GPT-4o: “Create a 1080x1350 advertising poster for the Donuts brand, with a beige background with the word donuts repeatedly in transparent, at the top written in brown uppercase ’ESTOU A ISTO’, below a chocolate donut and a hand holding it, thumb at the bottom and index at the top as if measuring the diameter, in the bottom left corner written in brown uppercase ’DE IR COMPRAR DONUTS’.”
6	AI vs. H	KitKat	GPT-4o: “Create a 1107x1536 advertising poster for Nestlé’s KitKat, red background, upper left corner a drawn red mug with ’NOVO’ written over in white with blue borders, centered upper part beside mug written in white ’Seu break favorito ficou’ with ’break’ highlighted, below in smoke shape ’Ainda Melhor’ coming out of transparent mug with chocolate drink, mug has KitKat brand and Nestlé logo, bottom right corner of KitKat logo says ’ACHOCOLATADO’ in white uppercase.”
7	AI vs. H	Mimosa	GPT-4o: “Create a 2048x818 advertising poster for the Mimosa brand, green background, on the left side a young girl with brown hair with a transparent glass of milk covering her mouth, on the glass a drawn smile and two pink circles as cheeks, on the right side the Mimosa logo and below written in white uppercase ’É PARTE DE NÓS.’ ”
8	AI vs. H	SuperBock	GPT-4o: “Create a 1080x1350 advertising poster for SuperBock, red background, top centered written in white uppercase ’É NATURAL QUE NOS SUBA À CABEÇA’, below a SuperBock bottle with foam coming out, beside bottle written in white ’Eleita a melhor cerveja sem glúten do mundo’ below ’WORLD BEER AWARDS STYLE WINNER WORLD’S BEST 2025’. At bottom small warning in white ’Sê responsável. Bebe com moderação. 5.2% álcool.’ ”
9	AI vs. H	Milka	GPT-4o: “Create a 1080x1080 advertising poster for Milka, purple background, top centered written in white uppercase ’O SABOR QUE VEM DOS’ and in blue highlighted style ’Alpes’, below a purple and white cow with red collar and yellow bell in a purple meadow, left side a mountain in purple tones. Bottom right corner white Milka logo.”

Continued on next page

Table S1 – continued from previous page

Pair	Contrast	Brand	AI Model & Exact Prompt
10	AI vs. H	Pizza Hut	GPT-4o: "Create a 1122x1402 advertising poster for Pizza Hut, red checkered background, top has a pizza cut into 8 slices on a napkin, bottom left corner a hand holding a slice, bottom right corner a hand holding a fork touching pizza, below pizza written in white uppercase 'OBRIGADO POR PARTILHARES A MESA CONNOSCO', below that Pizza Hut logo."
11	AI vs. H	Heinz	GPT-4o: [Exact prompt not recorded; the image was generated by a project collaborator with a product-focused prompt describing the Heinz campaign. This is the only undocumented stimulus.]
12	HAI vs. H	Compal	GPT-4o: "I want you to improve this image to make it more appealing to the public."
13	HAI vs. H	Gillette	GPT-4o: "I want you to improve this image to make it more appealing to the public."
14	HAI vs. H	Olá	GPT-4o: "I want you to improve this image to make it more appealing to the public."
15	HAI vs. H	Dodot	GPT-4o: "I want you to improve this image to make it more appealing to the public."
16	HAI vs. H	Donuts	GPT-4o: "I want you to improve this image to make it more appealing to the public."
17	HAI vs. H	KitKat	GPT-4o: "I want you to improve this image to make it more appealing to the public."
18	HAI vs. H	Mimosa	GPT-4o: "I want you to improve this image to make it more appealing to the public."
19	HAI vs. H	SuperBock	GPT-4o: "I want you to improve this image to make it more appealing to the public."
20	HAI vs. H	Milka	GPT-4o: "I want you to improve this image to make it more appealing to the public."
21	HAI vs. H	Pizza Hut	GPT-4o: "I want you to improve this image to make it more appealing to the public."
22	HAI vs. H	Heinz	GPT-4o: "I want you to improve this image to make it more appealing to the public."

Supplementary Materials: Complete Profiling Questionnaire

Table S2 presents all verbatim items from the screening and profiling questionnaire administered to participants via Microsoft Forms prior to the eye-tracking experiment.

Table S2: Complete inventory of verbatim questionnaire items, constructs, and response scales (administered in Portuguese).

#	Construct / Domain	Verbatim Item Text (Portuguese Original / English Translation)	Response Scale
Q1	Social Media Usage	Quantas horas por dia passa habitualmente nas redes sociais? *(How many hours per day do you usually spend on social media?)*	Categorical (< 1h, 1–2h, 2–4h, > 4h)
Q2	Platforms Used	Quais as plataformas que utiliza regularmente? *(Which platforms do you use regularly?)*	Multiple choice (TikTok, Instagram, YouTube, etc.)
Q3	Attention to Ads	Com que frequência repara ou presta atenção a conteúdos de marketing/publicidade nas redes sociais? *(How often do you notice marketing content on social media?)*	5-point scale (1 = Never to 5 = Always)
Q4	GenAI Usage Frequency	Com que frequência utiliza ferramentas de Inteligência Artificial Generativa? *(How often do you use Generative AI tools?)*	Frequency scale (Daily, Weekly, Monthly, Rarely, Never)
Q5	Known Text LLMs	Quais destas ferramentas de IA baseadas em texto conhece ou já utilizou? *(Which text-based AI tools/LLMs have you used?)*	Multiple choice (ChatGPT, Gemini, Claude, Copilot, etc.)
Q6	GenAI Purposes	Para que fins utiliza estas ferramentas? *(For what purposes do you use these tools?)*	Multiple choice (Academic, Work, Leisure, Content Creation)
Q7	AI Image Familiarity	Qual é o seu grau de familiaridade com imagens geradas por IA? *(How familiar are you with AI-generated images?)*	5-point scale (1 = Not familiar to 5 = Expert)
Q8	AI Image Generation	Já utilizou alguma ferramenta de IA especificamente para gerar ou editar imagens? *(Have you used AI tools specifically to generate or edit images?)*	Categorical (Yes frequently, Yes occasionally, No)
Q9	Exposure Frequency	Com que frequência encontra imagens geradas por IA no seu dia a dia? *(How often do you encounter AI-generated images?)*	Frequency scale (Daily, Often, Sometimes, Rarely, Never)
Q10	AI Discrimination	Como avalia a sua capacidade de distinguir uma imagem real de uma gerada por IA? *(How do you rate your ability to distinguish real vs. AI images?)*	5-point scale (1 = No ability to 5 = Easily distinguish)
Q11	AI Creativity	As imagens geradas por IA podem ser tão criativas quanto as criadas por humanos. *(AI-generated images can be as creative as those created by humans.)*	5-point Likert scale (1 = Strongly Disagree to 5 = Strongly Agree)
Q12	Ad Comfort	Sinto-me confortável ao ver imagens geradas por IA utilizadas em publicidade comercial. *(I feel comfortable seeing AI-generated images in commercial advertising.)*	5-point Likert scale (1 = Strongly Disagree to 5 = Strongly Agree)
Q13	Mandatory Disclosure	As empresas devem divulgar quando as suas imagens de marketing são geradas por IA. *(Companies must disclose when marketing images are AI-generated.)*	5-point Likert scale (1 = Strongly Disagree to 5 = Strongly Agree)
Q14	Perceived Authenticity	O conteúdo de marketing gerado por IA parece-me menos autêntico. *(AI-generated marketing content seems less authentic to me.)*	5-point Likert scale (1 = Strongly Disagree to 5 = Strongly Agree)
Q15	AI Ethics	Utilizar IA para criar imagens de marketing é eticamente aceitável. *(Using AI to create marketing images is ethically acceptable.)*	5-point Likert scale (1 = Strongly Disagree to 5 = Strongly Agree)
Q16	Marketing Interest	Qual o seu interesse geral por marketing e publicidade? *(What is your general interest in marketing and advertising?)*	5-point scale (1 = No interest to 5 = Very interested)
Q17	Visual Ad Attention	No seu dia-a-dia, qual é o seu nível de atenção a anúncios visuais? *(In daily life, what is your level of attention to visual ads?)*	5-point scale (1 = Ignore completely to 5 = Pay close attention)
Q18	First Visual Element	Ao visualizar um anúncio, que elemento costuma captar a sua atenção primeiro? *(When viewing an ad, what element usually catches your attention first?)*	Categorical (Colors/Contrast, Text, Human Faces, Product, Logo)
Q19	Impulse to Buy	Com que frequência sente vontade de comprar um produto apenas pelo anúncio? *(How often do you feel like buying a product based solely on the ad?)*	Frequency scale (Always, Often, Sometimes, Rarely, Never)
Q20	Visual Quality Impact	Já efectuou alguma compra influenciada principalmente pela qualidade visual de um anúncio? *(Have you made a purchase mainly influenced by an ad's visual quality?)*	Categorical (Yes, No, Not sure)
